# Tie-Over Bolster Pressure Dressing Improves Outcomes of Skin Substitutes Xenografts on Athymic Mice

**DOI:** 10.3390/ijms23105507

**Published:** 2022-05-14

**Authors:** Andréanne Cartier, Martin A. Barbier, Danielle Larouche, Amélie Morissette, Ariane Bussières, Livia Montalin, Chanel Beaudoin Cloutier, Lucie Germain

**Affiliations:** 1 Centre de Recherche en Organogénèse Expérimentale de l’Université Laval/LOEX, Department of Surgery, Faculty of Medicine, Université Laval, Quebec, QC G1V 0A6, Canada; andreanne.cartier.1@ulaval.ca (A.C.); martin-alexandre.barbier.1@ulaval.ca (M.A.B.); danielle.larouche@crchudequebec.ulaval.ca (D.L.); amelie.morissette@crchudequebec.ulaval.ca (A.M.); busse007@hotmail.com (A.B.); docteureliviamontalin@videotron.ca (L.M.); chanel.beaudoin-cloutier.1@ulaval.ca (C.B.C.); 2 Centre de Recherche, CHU de Québec-Université Laval, Regenerative Medicine Division, Quebec, QC G1J 1Z4, Canada; 3 Unité des Grands Brûlés, CHU de Québec-Université Laval, Quebec, QC G1J 1Z4, Canada

**Keywords:** tissue engineering, skin substitutes, skin graft, murine model

## Abstract

The efficacy of skin substitutes is established for the treatment of burn injuries, but its use is not limited to this condition. This technology has the potential to improve the treatment of various conditions by offering highly advanced and personalized treatments. In vivo studies are challenging but essential to move to clinical use in humans. Mice are the most widely used species in preclinical studies, but the main drawback of this model is the limited surface area of the graft in long-term transplantation studies caused by the displacement and the contraction of the graft. We improved the conventional surgical procedures by stabilizing the chamber covering the graft with intramuscular sutures and by adding a tie-over bolster dressing. The current study was therefore performed to compare outcomes of skin grafts between the conventional and optimized skin graft model. Human self-assembled skin substitutes (SASSs) were prepared and grafted to athymic mice either by the conventional method or by the new grafting method. Graft healing and complications were assessed using digital photographs on postoperative days 7, 14, and 21. Similar structure and organization were observed by histological staining. The new grafting method reduced medium and large displacement events by 1.26-fold and medium and large contraction events by 1.8-fold, leading to a 1.6-fold increase in graft surface area compared to skin substitutes grafted with the usual method. This innovation ensures better reproducibility and consistency of skin substitute transplants on mice.

## 1. Introduction

The skin is the largest organ of the human body and is prone to diverse types of impairment including wounds due to trauma, surgery, underlying medical conditions (such as diabetic foot ulcers), congenital skin disease such as epidermolysis bullosa or the most common cause of major skin loss, burn injuries [1,2,3]. When a significant portion of the deep dermis layer is affected, the epidermis is no longer able to self-regenerate and skin grafts are needed to cover the wound [4,5]. Moreover, healing capability can be impaired by several conditions such as vascular insufficiency, malnutrition, or infection, which leads to chronic wounds and to significant disability or even death [6]. Moreover, in the context of an ageing population with an increasing rate of diabetes and vascular diseases, chronic wounds and skin ulcers are rising [7,8,9]. Taken together, acute and chronic cutaneous wounds have major healthcare implications and account for more than 4% of the healthcare budget in industrialized countries, which stresses the importance of innovative treatments [10,11]. 

In response to a critical need for early permanent coverage for burn injuries with limited healthy donor tissue, a variety of skin substitutes have been developed in the last two decades [12,13,14]. Recently, bilayer substitutes have proved their efficacy for long-term coverage of full-thickness skin wounds in severely burned patients [15]. However, the need for skin substitutes is not limited to burn patients. Further studies are needed to extend their use in various conditions and to address key limitations of bioengineered skin such as the inability to reproduce skin appendages, and high manufacturing costs [16,17]. 

Research to develop bioengineered skin substitutes is complex, both scientifically and from a regulatory standpoint [18,19]. To move to first-in-human and early-stage trials, preclinical studies with in vivo evaluation need to be completed to reduce the risks of harm and to maximize the likelihood that the intervention will benefit humans. Since in vitro studies are unable to recreate the dynamic process of wound healing, in vivo experimentations are performed to test the efficacy of skin substitutes [20,21]. In vivo studies can assess parameters of the biological responses occurring in the patient upon grafting of the biomaterial such as angiogenesis, inflammatory responses, and interactions of the graft with the wound bed [22]. 

Mice are the most widely used species in preclinical wound healing studies because of their availability, low cost, and ease of handling [23,24]. One major advantage of rodents is the variety of immunodeficient strains available [25]. These models are essential to control the immune responses following xenotransplantation of human skin on mice to avoid graft rejection. Nevertheless, the translation of mouse models to humans remains a challenge due to the physiological and anatomical differences existing between these two species [23,26,27]. Rodents are “loose-skinned” mammals and have a thin layer of striated muscle located between the subcutaneous fat and dermis, the panniculus carnosus [28,29]. As a result, mice heal wounds mainly by contraction while humans heal by reepithelialization and granulation tissue formation. The contraction is problematic during reconstructed skin grafts since it can be strong enough that the surrounding mouse skin completely covers the graft which may hide the regenerative capacity of the grafted human skin substitute. Several methods and surgical devices have been developed to “humanize” the wound healing models in rodents.

The first technique described is the direct transplant model where a full-thickness excisional wound is created on the flanks of the mouse and a skin graft is transplanted onto the muscle in direct contact with the wound borders [30,31,32,33,34]. Numerous variations of these models exist to reduce contraction of host skin such as the removal of the panniculus carnosus, the use of opposing sutures, biological glue, and various conventional and biological wound dressings [23,35,36,37,38,39,40,41,42,43,44]. However, contraction and migration of the cells from wound borders cannot be avoided with these techniques. Alternatively, the splinting model approach sutures the wound edges into a silicone ring to force the wound to heal by reepithelialization [45,46,47]. This technique limits contraction but does not protect the surgical sites from environmental contamination and self-mutilation [18]. Several devices have been described varying in size, shape, and composition to alleviate these latter drawbacks [48]. The Fusenig chamber is a broadly used model that involves sewing a chamber onto the animal back to inhibit the contraction and growth of animal skin onto the grafted area and to encase the skin substitute safely [49,50,51,52]. Multiple devices have been designed varying in size, shape, and composition according to the need of the experiment and preferred animal species, but the basic principle of these models is the same: the lower part of the chamber creates an open wound area that is not in contact with the surrounding skin and the upper part of the chamber is a dome that protects the grafted area. Some authors combined the upper and lower parts of the chamber in one single device [53]. Alternatively, another version of the modified Fusenig’s chambers only required the lower part of the chamber to isolate the animal skin from the graft and applied wound dressing to cover the ring area [54,55,56]. Translucent variation in the chamber has also been developed for continuous monitoring of blood vessels and granulation tissue during in vivo periods [57,58,59]. Since the upper part of the chamber replaces the wound dressing, this model has been preferred over dressing models because some studies suggest that dressing may damage the graft and because it requires more manipulation with frequent renewals [60,61,62,63]. Despite the recent progress, the current experimental model does not perfectly mirror human skin grafts and studies are facing challenges to bridge the gap between preclinical and clinical studies. The main remaining weakness is the significant grafted surface reduction over time. This is a major limitation since the size of the graft is critical to evaluate graft survival and for research focusing on the reproduction of skin appendages. 

Another concern is to mimic as closely as possible procedures used for the skin graft in humans. In a clinical setting, pressure dressings are routinely used by surgeons during skin transplantation to secure the graft until it stabilizes; thus preventing common causes of skin graft loss: the formation of a hematoma under the graft, infection of the grafted skin, and shear forces at the interface [64]. These methods stand from the principles of successful graft take initially described by Blair and Brown in 1929. The more well-established method for fixing the graft to the wound bed and to secure the dressing to the graft is the tie-over bolster technique. In modern medicine, the latter is reserved for some recipient sites with two surfaces of free margin or sites with underlying structures where the placement of quilting sutures is difficult. However, some simple prevention measures used in humans to prevent skin graft complications such as patient early immobilization cannot be applied in rodents. Improved protocols in animal research have standardized the use of analgesia and anti-inflammatory drugs to minimize animal discomfort after surgery. As a result, the animal generally resumes its normal activity within an hour of the operation, which leads to a high risk of displacement and may compromise graft survival.

According to the principles of refinement, reduction, and replacement of humane experimental techniques of Russel and Burch [65], we aimed to refine the Fusenig chamber model in order to optimize conditions for long-term successful graft take and ultimately to reduce the numbers of animals used in future experiments. 

Our group has already used Fusenig’s chamber method with success to graft skin substitutes [49,66,67,68,69]. Here, we describe a new model that reduced the impact of contraction on grafts and prevents skin graft loss. In opposition to the conventional technique where the chamber is fixed to the surrounding skin, the new method involves intramuscular sutures in order to limit shear force transmission to the skin graft. Moreover, the addition of a tie-over bolster ensures a stable dressing that would resist any disturbance generated by the animal mobility. The current study was therefore performed to compare outcomes of human bilayered self-assembled skin substitutes xenografts between the conventional and optimized skin graft model.

## 2. Results

### 2.1. Mean Surface Area of SASS Grafts Is Larger with the New Technique at Any Time Points Postoperatively 

Experiments were designed to define the importance of specific surgical parameters during the transplantation of human skin substitutes on mice. A total of 37 athymic mice were divided into two groups to define the best surgical condition (11 in the control group and 22 in the experimental group). As controls, skin substitutes were grafted with the standard technique of our mouse model using the Fusenig’s chamber as previously published [49]. The purpose of Fusenig’s chamber is to minimize contraction and contribution of the surrounding mouse skin to wound healing. This chamber allows to better assess the regenerative potential of the grafted human skin substitute. The experimental group was grafted with the new surgical model which differs from the previous model by the addition of intramuscular sutures to stabilize the Fusenig’s chamber and by inserting tie-over bolster dressing to immobilize the graft inside the chamber. The tie-over bolster dressing from the experimental group was removed between the 6th and 10th postoperative days. Thereafter, the grafts were protected exclusively with the upper part of the Fusenig’s chamber since grafts adhered to the graft bed and are less susceptible to sheering forces at this stage. The graft take was assessed clinically on days 7, 14, and 21, at which point photographs were taken to document the graft take, displacement, and contraction. ImageJ software was used to assess the size of the graft from pictures taken during the follow-up. Animals were euthanized 21 days post-transplantation and grafts were excised for histological analysis. 

**Figure 1:**
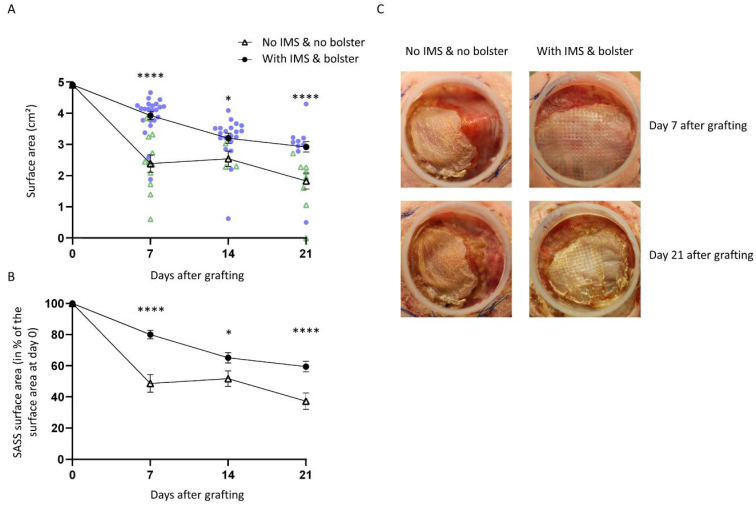
SASS surface area over time after grafting. (**A**) Mean surface area of SASS grafts is improved when the Fusenig’s chamber is secured with intramuscular sutures (IMS) and a bolster dressing is added over the SASS compared to only sutured to mouse skin. N = 2 to 3; n = 3 to 22. Each black point represents the mean and vertical error bars represent the standard error of the mean (SEM). Blue points represent each data point for skin substitutes grafted with IMS and bolster whereas green triangles represent each data point for skin substitutes grafted without IMS and without bolster. (**B**) Surface area of SASSs after grafting, expressed as a percentage of the SASS surface area before grafting. Each black point represents the mean and vertical error bars represent the standard error of the mean (SEM). (**C**) Representative examples of macroscopic pictures of SASSs grafted on the back of athymic mice with the conventional method (without IMS and without bolster dressing) or with IMS and a bolster. Statistical analyses were performed using Mann–Whitney’s non-parametric test. * *p* < 0.05; **** *p* < 0.0001.

When using the intramuscular sutures and the tie-over bolster dressing we measured a mean surface area of SASSs of 3.93 (±0.13) cm^2^, 3.3 (±0.16) cm^2^, and 2.92 (±0.17) cm^2^ at 7, 14, and 21 days, respectively (mean ± SEM). Without these stabilization measures, we observed with the conventional technique a mean surface area of SASSs of 2.39 (±0.28) cm^2^, 2.54 (±0.25) cm^2^, and 1.83 (±0.26) cm^2^ at 7, 14, and 21 days, respectively. Our results demonstrate that the new method results in a larger surface area of the graft at any time points postoperatively (Figure 1A). Moreover, 21 days after grafting without IMS or bolster, the grafted SASSs area was 37% of the original graft whereas with the new method the SASSs area was 59% of the original graft (Figure 1B), which corresponds to a 1.6-fold increase. Representative examples are shown in Figure 1C. 

### 2.2. The Use of Tie-Over Dressing and Intramuscular Sutures Significantly Reduces SASS Grafts Displacement

**Figure 2:**
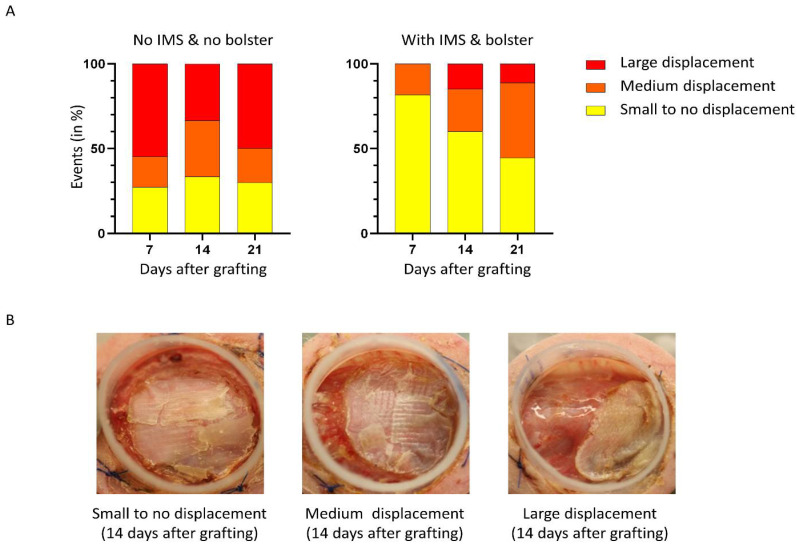
Qualitative assessment of displacement events following surgery. (**A**) Displacement of the graft was assessed over time after grafting. For each mouse, the displacement was evaluated (large, medium, or small to no displacement). Results are presented as the percentage of mice in each category 7, 14, and 21 days after grafting. (**B**) Representative examples of SASS displacement 14 days after grafting on the back of athymic mice.

For proper take of skin grafts, the graft needs to be adherent to the wound bed and immobilized for the first few days. Both the tie-over bolster dressing and the intramuscular sutures can help achieve this by minimizing shear and traction to the healing skin graft. To evaluate the advantages of the new method in preventing skin graft displacement, we used a severity scale (Figure 2B large, medium, or small to no displacement) and assessed macroscopically the importance of displacements over time for each group. When adding our new surgical manipulations, we observed very few displacements of the graft with a rate of large displacement of 0%, 15%, and 11% at 7, 14, and 21 days, respectively. On the other hand, a high rate of large displacement was observed with the conventional method with 54%, 33%, and 50% at 7, 14, and 21 days, respectively. The most significant difference between the groups was observed during the first postoperative assessment on day 7 (Figure 2A).

In the group with no IMS and no bolster, most displacements occurred before day 7 after which the event rate was fairly stable. Interestingly, displacements occurred later in the group with IMS and bolster, between days 7 and 14 which corresponds to the period following the withdrawal of the bolster. Our results demonstrate that displacements occurred mainly after removing the bolster which highlights the strong protective effect of the tie-over bolster dressing and sutures on graft displacement. In the group with no IMS and no bolster, the displacements were relatively stable at each time point, indicating that displacements occur mainly in the first days after transplantation when the skin graft is not strongly adhered yet to the wound bed.

### 2.3. The Use of Tie-Over Dressing and Intramuscular Sutures Significantly Reduces Contraction of SASS Grafts 

**Figure 3:**
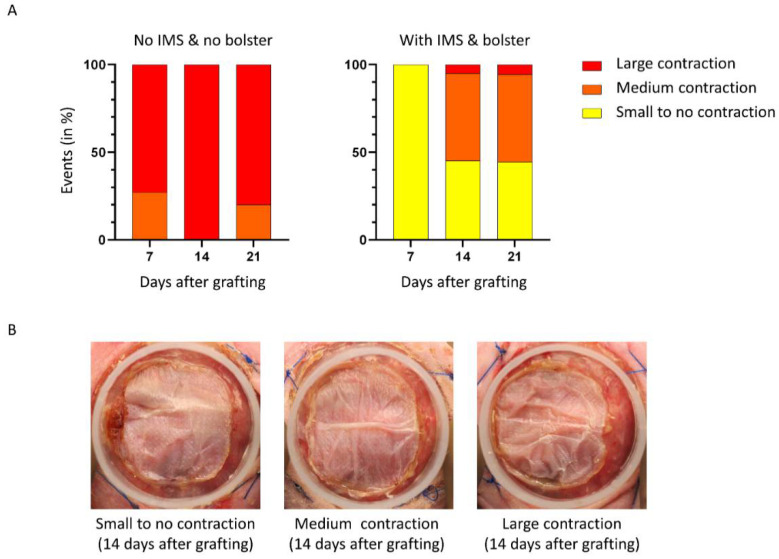
Qualitative assessment of contraction events following surgery. (**A**) Contraction of the graft was assessed over time after grafting. For each mouse, the contraction was evaluated (large, medium, or small to no displacement). Results are presented as the percentage of mice in each category 7, 14, and 21 days after grafting. (**B**) Representative examples of SASS contraction 14 days after grafting on the back of athymic mice.

The second main cause of loss of surface area is the contraction of graft. We chose to define contraction as the shrinkage of the skin graft. To determine if the use of IMS and bolster have an effect on this parameter, we qualitatively assessed contraction in each group, using a severity scale (Figure 3B large, medium, or small to no contraction) at different time points. 

As shown in Figure 3A, we observed a higher rate of large and medium contraction in the group without IMS and bolster at all time points postoperatively compared to the group grafted with the new method. Most contractions of skin graft occurred a few days after grafting (73% of large contractions at day 7 in the control group). Moreover, the highest decrease in surface area occurred between days 0 and 7 in that group (Figure 1A). In contrast, SASSs grafted with IMS and bolster showed a lower number of large contraction events during the first week (0% and 0% of large and medium contractions at day 7, respectively), but a delay in the initiation of contraction with the greatest contraction occurring between days 7 and 14 (5% and 50% of large and medium contractions, respectively, at day 14). The decrease in surface area was fairly constant between days 0 and 14 after grafting in that group (Figure 1A). However, the contraction was continuous over time since it progressed in both groups on subsequent observations. According to our results, the use of IMS and bolster are effective in minimizing graft contraction highlighting the importance of securing the graft to prevent common causes of skin graft loss such as displacement and contraction.

### 2.4. Hematoxylin and Eosin Staining Showed No Significant Difference between the Two Methods in the Histological Analysis of the SASS Grafts

**Figure 4:**
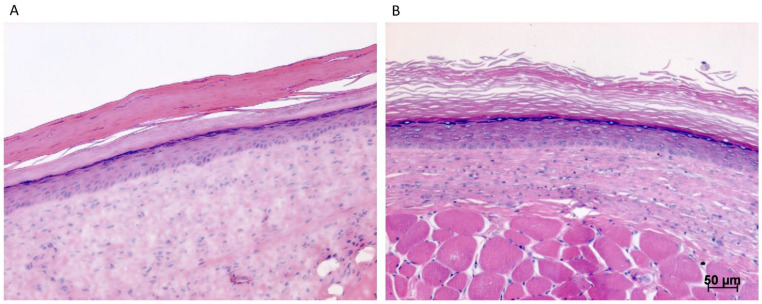
Hematoxylin and eosin staining of SASSs harvested 21 days after grafting on the back of athymic mice. (**A**) Skin substitute grafted without IMS and bolster. (**B**) Skin substitute grafted with IMS and bolster.

**Figure 5:**
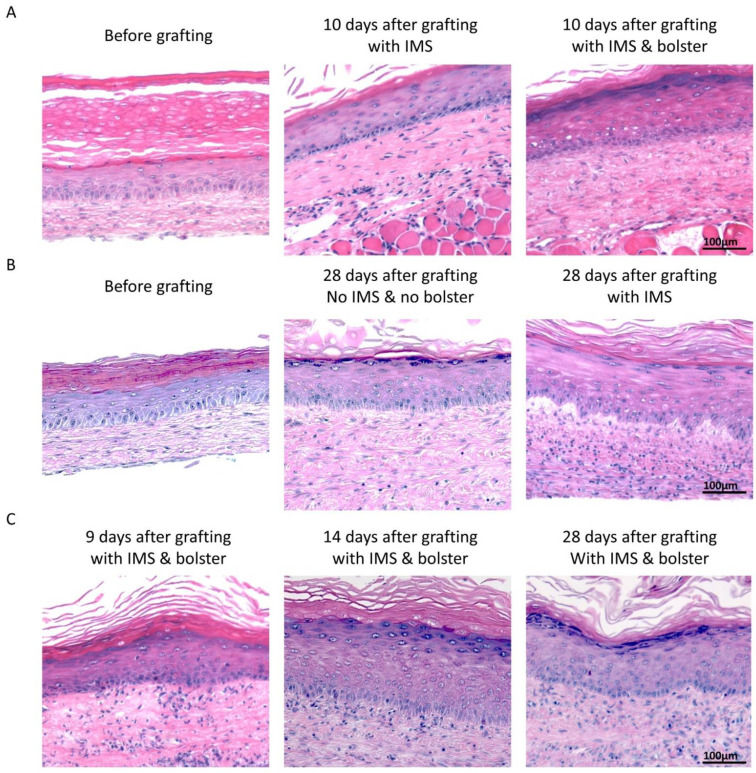
Hematoxylin and eosin staining of reconstructed skins after grafting. (**A**) SASSs were grafted with IMS or with IMS and a bolster and harvested after 10 days. (**B**) SASSs were grafted with the conventional method (no IMS and no bolster) or with both and harvested after 28 days. (**C**) SASSs were grafted with IMS and a bolster, and harvested at different time points (10, 14, and 21 days).

Animals were euthanized between 9 and 28 days postoperatively and skin graft biopsies were harvested. Histological analysis with hematoxylin and eosin staining confirmed the survival of engrafted SASS skin substitutes (Figure 4 and Figure 5). The expected epidermal layers (stratum basale, spinosum, granulosum, and corneum) are present before grafting and at all time points after grafting (Figure 5). The number of cell layers of the grafted reconstituted skin is consistent with human skin, being greater than that of mouse skin.

When comparing the histology in successfully grafted areas, no difference was observed in the architecture and organization of epidermis and dermis between the two methods (Figure 4 and Figure 5). There was no difference either for the histology of SASSs after grafting with IMS only or with IMS and a bolster (Figure 5). 

## 3. Discussion

Grafting cultured skin substitutes is a complex process, with high requirements for stringency to be successful consistently. The lack of an ideal animal model that replicates the healing process of humans limits the accuracy and applicability in testing the effectiveness of novel skin substitutes therapies. Since mice are the most widely used animals for in vivo studies in this area, a broad spectrum of methodological variation is described with their own limitations. The main limitation is the significant grafted surface reduction over time. The decrease in graft surface area is most often attributed to contraction observed in mice as the primary healing mechanism. The necessity for a technique that provides grafts of reproducible size in a standardized but rapid manner remains. 

Our group has already used Fusenig’s chamber method with efficacy to graft skin substitutes over the last three decades. We have observed that graft take and the decrease in the size of the grafts can also be explained by other mechanisms such as the formation of hematoma or seroma under the graft, displacement of the graft, or by shear or traction injury that disrupts skin graft healing. These complications are also observed during skin transplantation in humans, although several precautions are used to avoid them. Animal model validity is discussed in terms of the similarity between the model and the human condition. In humans, skin grafts are overlaid with compressive dressing until postoperative days 5–10 to secure the skin graft during the stage of wound healing: imbibition, inosculation, revascularization. Before this period, the transplant is not integrated into the wound bed and is more at risk of complications that can affect graft take. 

In this study, we investigated the influence of intramuscular sutures to fix the Fusenig’s chamber, and of tie-over bolster dressing in our in vivo mouse transplantation model. We successfully demonstrated that the novel method increases the surface area of the skin graft by limiting displacement and contraction over time. Our results support the intuitive postulate that for proper take of skin graft, the graft needs to be stable and adherent to wound bed for the first few days. Even if the traditional tie-over dressing is commonly used in clinical settings, this technique has been debated since some authors have reported that pressure is not required for a successful graft [64,70,71]. However, we believe that this type of dressing is necessary when grafting onto mice since our recipient, the bony spinal area, is unstable due to mouse movements which cannot be limited. Our preliminary experiments assessed the effect of bolus dressing and intramuscular stitches separately and showed that the size of the grafts was increased in both groups (data not shown). Therefore, we decided to incorporate these two manipulations into our new model.

The use of Fusenig’s chamber is essential to encase skin substitutes and prevent contraction of the mouse’s surrounding skin. The use of other devices has also been described to limit contraction, but the size of the analyzed tissues is markedly limited given the dimensions of these devices. Since the final size of the skin graft obtained after transplantation depends not only on the skin graft take but also on the initial size of the skin substitutes. It is important to have devices that allow larger transplants since the size of the graft is critical to evaluate graft survival over a long period of time and for working on the reproduction of skin appendages. Some authors use very small domes that do not require suture fixation on the principle that such fixation can induce inflammation in the local area and allow more contraction [53]. The stitches used in our new technique are intramuscular sutures; therefore, we can stipulate that they induce more inflammation than cutaneous sutures used in the conventional method. However, our results show a decreased contraction for the novel grafting method. 

One difference between human and mouse skin is its mobility. Mouse skin is not attached to the underlying tissues like human skin. The loose mouse skin allows great mobility of the chamber when it is attached only to the skin. It puts the graft at risk of shear or traction injury by the silicone chamber. These complications can be prevented with a better fixation of the chamber to prevent its displacement such as with intramuscular stitches which justifies their use in our protocol. We observed a rate of medium to large contraction of 100% in the control group compared to only 55.5% in the experimental group 21 days after grafting. When the surface area is evaluated according to the initial size of the graft, we observe that 21 days after grafting SASSs grafted with IMS and bolster have a surface area of 59.5% of the graft at day 0 compared to 37.2% without IMS and without bolster, which represents a 1.6-fold increase in surface area.

Our results demonstrated that the inflammation induced by more invasive sutures does not translate into an increase in contraction, contrary to what has been described in the literature previously. Moreover, we obtained lower contraction rates than what is described in the literature [47,72,73,74]. 

The prevention of postoperative graft displacement is crucial for the success of skin grafting. In the control group, we observed a rate of large displacement of 50% compared to 11.1% in the experimental group. Displacements significantly affect the graft surface areas because the graft can be folded back on itself and several parts are no longer in contact with the wound bed. Moreover, the absence of stability prevents attachment and thus affects initial graft take. Our method, using tie-over bolster dressing and IMS seems to be ideal for fixing the graft onto the graft bed, even though this procedure is more time-consuming than methods in which the graft is simply placed onto the graft bed. To address this problem, previous groups have used sutures or glue directly under the graft [75]. Unlike these techniques, our method has the advantage of protecting the graft without direct manipulation of the graft itself which risks damaging the transplant. Moreover, the tie-over bolster dressing technique not only stabilizes the graft but also absorbs exudate from the wound. In some situations, such as wound infection, the exudates can be very abundant in the chamber and harmful to the skin graft. The tie-over bolster dressing is an interesting tool that allows early recognition of these situations by monitoring the color of the gauze. If the original white color has completely faded, the tie-over dressing should be removed and replaced. Bolster dressings had already been used in studies of skin grafting in mice, but these techniques had been abandoned since some authors had described that they could damage the skin substitutes. We observed an imprint of the mesh structure of Adaptic gauze onto the skin substitutes but histological analysis demonstrated that it did not affect the epidermal structure or biological viability and only lasted for a few days after the bolster and Adaptic gauze were taken out. 

Concerning the limitations of the study, the new technique is technically more complex than the conventional technique which may limit its accessibility for less experienced technicians. The complexity of manipulations and additional steps to the procedure increases the time of surgery and anesthesia which increases recovery time and the risk of dehydration. However, this time difference decreases with the technicians’ learning curve. The bolus dressing can easily be applied following the cessation or decrease of anesthesia to reduce the exposure of the mouse. Moreover, intramuscular stitch placement increases the risk of complications from deep tissue manipulation of the mouse (hemorrhagic risk and the risk of deep viscera perforation (pneumothorax, enterotomy)). However, this risk is low, and is reduced when the procedure is performed by experienced technicians. The bolus dressing must be left in place for a minimum of 5 days, which limits graft observations during this period. We did not test the new method for autologous cultured keratinocyte graft. Further studies need to be completed with the new techniques to be validated as a reliable model for autologous cultured keratinocyte graft.

The presented method limits contraction and displacement of the transplanted graft which increases the final size of the graft and sets optimized conditions for long-term successful graft take and follow-up which will ultimately favor the reduction in the number of animals used in future experiments. Moreover, this protocol ensures highly reproducible results. Taken together, these characteristics of our model offer many possibilities to study novel therapeutic strategies in the field of tissue engineering and regenerative medicine.

## 4. Materials and Methods

### 4.1. Isolation of Human Skin Fibroblasts and Keratinocytes

Skin biopsies were obtained from healthy patients undergoing elective surgery. Fibroblasts were extracted from newborn foreskin (1 year old) or adult breast skin (18 and 38 years old). Keratinocytes were extracted from newborn foreskin (1 year old) or adult breast skin (8 and 37 years old), as previously reported (Sergio et al., 2019, Goyer et al., 2019). Briefly, skin samples were washed 10 times in phosphate buffered saline (PBS) supplemented with 25 µg/mL gentamicin, 100 IU/mL penicillin G (Sigma-Aldrich, St. Louis, MO, USA), and 0.5 µg/mL fungizone. Samples were then cut into 2 × 0.5 cm^2^ strips and submitted to enzymatic digestion through overnight incubation in 500 µg/mL thermolysin in HEPES buffer (10 mM of 4-(2-hydroxyethyl)-1-piperazine ethane sulfonic acid (MP Biomedicals Inc., Solon, OH, USA), 6.7 mM potassium chloride, 142 mM sodium chloride, and 1 mM calcium chloride) at 4 °C. The next day, the epidermis was manually peeled off using pliers. The epidermis was digested for 30 min in trypsin-EDTA 0.05% *w*/*v* porcine trypsin 1-250 (Thermo Fisher Scientific, Ottawa, ON, Canada), 0.01% *w*/*v* EDTA (J.T. Baker), 2.8 mM D-glucose (EMD Millipore, Burlington, MA, USA), 100 IU/mL penicillin G, 25 μg/mL gentamicin, and phenol red (J.T. Baker) in PBS at 37 °C on a stirring plate. The dermis was digested for 3 h with 0.125 U/mL collagenase H (Roche Diagnostics, St. Louis, MO, USA) in Dulbecco’s Modified Eagle Medium (DMEM; Thermo Fisher Scientific, Ottawa, ON, Canada) supplemented with 10% *v*/*v* fetal bovine serum (FBS; Seradigm), 100 IU/mL penicillin G (Sigma-Aldrich, St. Louis, MO, USA), and 25 μg/mL gentamicin (Galenova, Saint-Hyacinthe, QC, Canada), on a shaker. Digestion of both epidermis and the dermis were stopped by doubling the volume of the suspension with the appropriate culture media. 

### 4.2. Human Feeder Layer

Human newborn foreskin fibroblasts were irradiated at 6000 rads and seeded at 8000 cells/cm^2^ in cell-culture-treated flasks in 0.26 mL/cm^2^ of keratinocyte culture media (ckDMH) consisting of three parts DMEM and one part Ham’s F12 medium (Thermo Fisher Scientific, Ottawa, ON, Canada) supplemented with 3.07 g/L NaHCO3 (J.T. Baker), 24.3 mg/L adenine (Sigma-Aldrich, St. Louis, MO, USA), 5% *v*/*v* inactivated FetalClone II serum (HyClone, Logan, UT, USA), 5 µg/mL insulin (Sigma-Aldrich, St. Louis, MO, USA), 0.4 µg/mL hydrocortisone (Galenova, Saint-Hyacinthe, QC, Canada), 0.212 µg/mL isoproterenol hydrochloride (Sigma-Aldrich, St. Louis, MO, USA), 10 ng/mL epidermal growth factor (Austral Biologicals, San Ramon, CA, USA), 100 UI/mL Penicillin G (Sigma-Aldrich, St. Louis, MO, USA), 25 µg/mL gentamicin (Gemini Bio, West Sacramento, CA, USA). Culture media was conditioned for 5–7 days prior to seeding keratinocytes. 

### 4.3. Monolayer Culture of Primary Human Fibroblasts and Keratinocytes

Primary fibroblasts were seeded at 4000 cells/cm^2^ in a cell-culture-treated flask, with 0.2 mL/cm^2^ fibroblast culture media (fDME) consisting of DMEM supplemented with 10% fetal bovine serum (FBS; Seradigm, VWR, Montreal, QC, Canada), 100 IU/mL penicillin G (Sigma-Aldrich, St. Louis, MO, USA), and 25 μg/mL gentamicin (Galenova, Saint-Hyacinthe, QC, Canada). Fibroblasts were trypsinized and passaged when they reached 90–100% confluency. Primary keratinocytes were seeded at 6667 cells/cm^2^ in cell-culture-treated flasks containing the human feeder layer and ckDMH media conditioned for 5–7 days. Cells were trypsinized and passaged when they reached 80–95% confluency.

### 4.4. Self-Assembled Skin Substitute Production

Human fibroblasts were seeded at 4000–20,000 cells/cm^2^ in a cell-culture-treated Petri dish (Falcon) containing a custom paper frame (Ahlstrom grade 237 filter) and held in place with metal ingots. Fibroblasts were cultured in fDME supplemented with 50 µg/mL ascorbic acid (Sigma-Aldrich, St. Louis, MO, USA) to promote extracellular matrix polymerization. Culture media was changed three times a week for 21–28 days, after which they form a manipulable fibroblast sheet. Human keratinocytes were seeded on top of a fibroblast sheet at 150,000 cells/cm^2^ in ckDMH supplemented with 50 µg/mL of ascorbic acid. After 4 days, 1–2 fibroblast sheets were superimposed on the bottom of the keratinocyte-containing fibroblast sheet and secured in place with ligating clips (Ethicon Inc., Cincinnati, OH, USA) to form a self-assembled skin substitute (SASS). SASSs were raised at the air–liquid interface on a polypropylene membrane (Spectral/Mesh; Spectrum Labs) and a custom steel frame. SASSs were then cultured for 10–21 days in ckDMH supplemented with 50 µg/mL of ascorbic acid, exempt of epidermal growth factor.

### 4.5. Skin Substitutes Preparation for Grafting

The bottoms (dermal side) of the SASSs were rinsed twice in DMEM supplemented with 100 IU/mL penicillin G (Sigma-Aldrich, St. Louis, MO, USA) and 25 μg/mL gentamicin (Galenova, Saint-Hyacinthe, QC, Canada). Then, 25 × 25 mm^2^ squares were cut with a scalpel and overlayed with a non-adhering dressing (Adaptic^TM^, Acelity, Mississauga, ON, Canada) secured in place with ligating clips for transportation. Ligating clips were removed with scissors before grafting.

### 4.6. Fusenig’s Chamber

**Figure 6:**
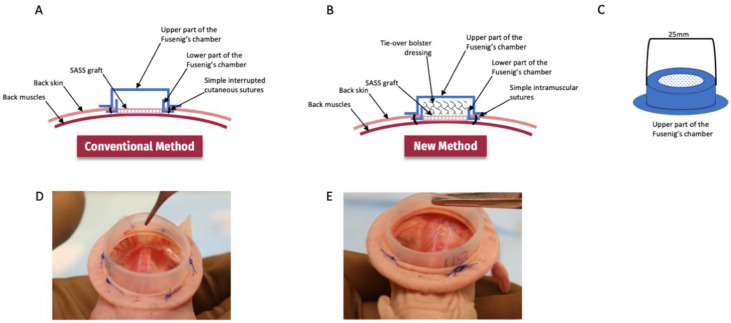
Schematic view of the two models studied. (**A**) The conventional method included the fixation of the chamber directly to the skin by simple interrupted cutaneous sutures and no additional dressing is added on the skin graft in the Fusenig’s chamber. (**B**) The new method included intramuscular sutures for the fixation of the Fusenig’s chamber and the addition of a tie-over bolster dressing to cover the skin graft. (**C**) Diagram of the upper part of the Fusenig’s chamber which is added after grafting to protect the graft. (**D**) Without intramuscular stitches the Fusenig’s chamber is not properly secured whereas with (**E**) intramuscular stitches, the chamber is tightly secured to the muscles limiting displacement.

Medical grade silicon, Nusil MED 4515 (Nusil, Carpinteria, CA, USA) was inserted into custom made molds (Figure 6) using a hydraulic press and heated at 139 °C for 90 min after which silicon chambers were extracted from the molds. A 2 cm diameter circle was cut from the top of the cap’s chamber and replaced with filter paper, secured with a silicon-based glue. Fusenig’s chambers were autoclaved before use.

### 4.7. Skin Substitutes Grafting on CD1-Foxn1nu Mice

**Figure 7:**
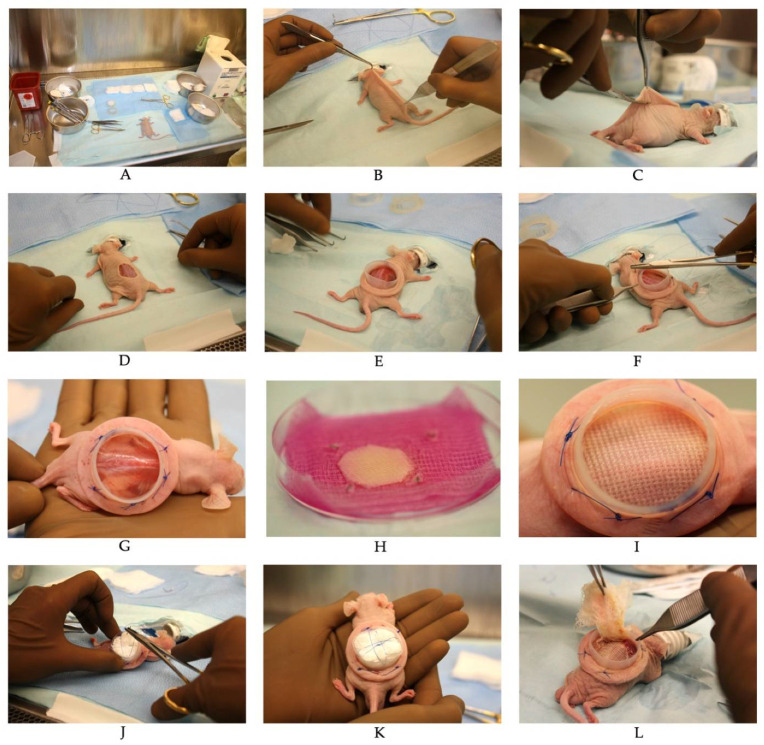
Grafting of SASSs on athymic mice. (**A**) Surgical procedures are performed in a biological safety cabinet with aseptic technique, and all materials are sterile. (**B**) The dorsal skin is pulled from the midline with forceps; (**C**,**D**) 3 cm^2^ of skin is excised to produce a full-thickness skin excisional wound; (**E**) the base of the silicon Fusenig’s chamber is inserted into the wound; (**F**,**G**) intramuscular sutures are placed to fix the Fusenig’s chamber on the mouse. (H) The SASS overlayed with the non-adhering dressing is prepared for grafting by cutting out the metal clips used to secure the SASS to the dressing during transportation to surgical room. (**I**) The SASS and the non-adherent dressing are placed together inside the Fusenig’s chamber, directly on the muscle. (**J**,**K**) Sterile gauze is placed on top of the Vaseline gauze and the tie-over threads are passed from inside out of the base of the Fusenig’s chamber to pull the appropriate tension to keep the dressing in place. (**L**) The tie-over bolster dressing is removed 7–10 days after surgery.

Six-week-old male athymic mice (Crl:CD1-Foxn1n; Charles River Laboratories, Laval, QC, Canada) were used as graft recipients and maintained under sterile housing conditions throughout the experiments. Mice were injected with 30.75 mg/kg ceftazidime (Teligent Inc., Buena, NJ, USA) prior to grafting as well as 24 and 48 h after surgery; 20 mg/kg carprofen (Zoetis Canada Inc., Kirkland, QC Canada) immediately after surgery. Mice were anesthetized by inhalation of 1.5–3% isoflurane (Baxter, Mississauga, ON, Canada) with 1 mg/kg buprenorphine-SR (Chiron Compounding Pharmacy Inc., Guelph, ON, Canada). A 3 cm^2^ portion of the mid back skin was excised. To promote vascularization of the graft, the fascicular panniculus was removed with pliers. A silicon Fusenig’s chamber (5 cm^2^ inner surface area) was inserted between the muscle and the skin to prevent reepithelialization of the wound by mouse skin and graft tampering by the mouse (Figure 6). The base of the chamber was secured in place with polypropylene thread (Prolene 4-0; Ethicon Inc., Cincinnati, OH, USA) through 4 intramuscular stitches (optimized method) or 4–6 stitches to mouse skin (conventional method). SASSs overlayed with the non-adhering dressing were then placed carefully on the exposed back muscle within the Fusenig’s chamber. For the new method, sterile gauzes were placed inside the Fusenig’s chamber as a bolus dressing to prevent graft displacement. Polyprolene thread (Prolene 4-0; Ethicon Inc., Cincinnati, OH, USA) was used to put pressure on the gauzes (Figure 7). After 7–10 days, 0.9% NaCl was poured inside the Fusenig’s chamber. Then, 2–5 min later, the bolus dressing and the non-adherent gauze were carefully removed from the graft.

### 4.8. Graft Area Analysis

Grafts were photographed weekly. The surface area was determined using ImageJ [76]. The contraction of the grafts was qualitatively assessed over time after grafting. For each mouse, the contraction was qualitatively evaluated and assigned to one of the three categories (large, medium, or small to no contraction). Results are presented as the percentage of mice in each category 7, 14, and 21 days after grafting. The displacement of the graft was qualitatively assessed over time after grafting. For each mouse, the displacement was qualitatively evaluated and assigned to one of the three categories (large, medium, or small to no displacement). Results are presented as the percentage of mice in each category 7, 14, and 21 days after grafting. Representative examples of each category are presented in Figure 2B and Figure 3B. Displacement and contraction qualitative assessment were performed in a blinded fashion by 3 evaluators and the median was used.

### 4.9. Histological Analysis

At the end of the experiment (days 9–28 after grafting), SASS samples were fixed overnight in 10% formalin and embedded in paraffin. Then, 5 µm sections were cut and stained with hematoxylin and eosin (HE). 

### 4.10. Statistical Analysis

Statistical analysis was performed using GraphPad Prism version 9.0.0 for Windows [77]. Surface area at days 7, 14, and 21 were analyzed using Mann–Whitney’s non-parametric test and confirmed using a linear mixed model.

## Data Availability

The data presented in this study are available upon request to the corresponding author.
